# Amusing Age Table

**Published:** 1857-11

**Authors:** 


### CURIOUS TABLE OF FIGURES.

JUST hand this table to the lady, and request her to tell you in which column or columns her age is contained. Add together the figures at the top of the columns in which her age is found, and you have the great secret. Thus: suppose her age to be seventeen. You will find the number seventeen only in two columns, viz., the first and fifth, and the first figures of these columns make seventeen. Here is the magic table:

| | | | | | |
|---|---|---|---|---|---|
| 1 | 2 | 4 | 8 | 16 | 32 |
| 3 | 3 | 6 | 9 | 17 | 33 |
| 5 | 6 | 6 | 10 | 18 | 34 |
| 7 | 7 | 7 | 11 | 19 | 35 |
| 9 | 10 | 12 | 12 | 20 | 36 |
| 11 | 11 | 13 | 13 | 21 | 37 |
| 13 | 14 | 14 | 14 | 22 | 38 |
| 15 | 15 | 15 | 15 | 23 | 39 |
| 17 | 18 | 20 | 24 | 24 | 40 |
| 19 | 19 | 21 | 25 | 25 | 41 |
| 21 | 22 | 22 | 26 | 26 | 42 |
| 23 | 23 | 23 | 27 | 27 | 43 |
| 25 | 26 | 28 | 28 | 28 | 44 |
| 27 | 27 | 29 | 29 | 29 | 45 |
| 29 | 30 | 30 | 30 | 30 | 46 |
| 31 | 31 | 31 | 31 | 31 | 47 |
| 33 | 34 | 36 | 40 | 48 | 48 |
| 35 | 35 | 37 | 41 | 49 | 49 |
| 37 | 38 | 38 | 42 | 50 | 50 |
| 39 | 39 | 39 | 43 | 51 | 51 |
| 41 | 42 | 44 | 44 | 52 | 52 |
| 43 | 43 | 45 | 45 | 53 | 53 |
| 45 | 46 | 46 | 46 | 54 | 54 |
| 47 | 47 | 47 | 47 | 55 | 55 |
| 49 | 50 | 52 | 56 | 56 | 56 |
| 51 | 51 | 53 | 57 | 57 | 57 |
| 53 | 54 | 54 | 58 | 58 | 58 |
| 55 | 55 | 55 | 59 | 59 | 59 |
| 57 | 58 | 60 | 60 | 60 | 60 |
| 59 | 59 | 61 | 61 | 61 | 61 |
| 61 | 62 | 62 | 62 | 62 | 62 |
| 63 | 63 | 63 | 63 | 63 | 63 |

If the above table should amuse boys from the street for an evening or two, and should make home more tolerable to some restless, gad-about girl, who is never happier than when flaunting on the street, or discoursing small talk with dandified young men at the theatre or opera, or tawdry eating saloon, we will feel repaid for its insertion: it amused us hugely in "sunnier days departed."

